# One-Step Reduction and Surface Modification of Graphene Oxide by 3-Hydroxy-2-Naphthoic Acid Hydrazide and Its Polypropylene Nanocomposites

**DOI:** 10.3390/nano7020025

**Published:** 2017-01-24

**Authors:** Xiang-Nan Xu, Xiao-Na Guan, Hui-Hua Zhou, Yue-Feng Zhu

**Affiliations:** 1 School of Materials Science and Engineering, Tsinghua University, Beijing 100084, China; xxn14@mails.tsinghua.edu.cn (X.-N.X.); gxn16@mails.tsinghua.edu.cn (X.-N.G.); zhouhh@tsinghua.edu.cn (H.-H.Z.); 2 Key Laboratory for Advanced Materials Processing Technology, Ministry of Education, Beijing 100084, China; 3 National Center for Electron Microscopy Beijing, Beijing 100084, China

**Keywords:** graphene, reduction, surface modification, nanocomposites

## Abstract

3-Hydroxy-2-naphthoic acid hydrazide (HNH), a new reductant and modifier, was applied to reduce and modify graphene oxide (GO) in a one-step process. The obtained HNH reduced graphene oxide (HNH-rGO) was characterized by X-ray diffraction (XRD), scanning electron microscope (SEM), Raman spectroscopy, X-ray photoelectron spectroscopic (XPS) and Fourier transform infrared spectra (FTIR). The results demonstrated that GO was successfully reduced to graphene and the surface of HNH-rGO was grafted with HNH. The interlayer space was increased from 0.751 nm to 1.921 nm, and its agglomeration was much more attenuated compared with GO. HNH-rGO/polypropylene and graphene/polypropylene composites were synthesized through melt-blending method. The viscosity was enhanced with increased addition of graphene and surface modified graphene demonstrated stronger rheological behavior improving effect than the untreated graphene.

## 1. Introduction

Graphene, a honeycomb quasi-two-dimensional material formed by sp^2^ hybrid carbon atoms, was firstly obtained by Geim and Novoselov in 2004 [1]. Since then, graphene (G) has drawn more and more attention in the applications of electrode material [2], biological composite material [3], liquid crystal materials [4,5], supercapacitor [6,7], polymer nanocomposites [8] as well as solar cells [9]. This is entirely based on its excellent properties such as good thermal and electrical conductivities, large theoretical specific surface area, flexibility, high intrinsic mobility and chemistry stability [10,11]. To produce graphene sheets, there already exist various effective methods, for example, mechanical exfoliation [1], chemical vapor deposition (CVD) [12,13,14,15], oxidation-reduction method [16,17], unzipping of carbon nanotubes [18,19,20], electrochemical method [21,22] and crystal epitaxial growth method [23,24] and so on.

It is its many intriguing properties that attract plenty of researchers to study graphene and make graphene polymer nanocomposites a hotspot in composite materials field. However, the strong intermolecular force between graphene sheets restricts the homogeneous dispersion within the polymer matrix. To avoid agglomeration of graphene sheets in a polymer matrix, modifications of graphene are urgently needed as essential methods. Covalent and noncovalent modifications are two approaches to modify and functionalize graphene [25]. Covalent modification is able to make modified graphene and polymer matrix connected through covalent bond, resulting in powerful capacity to bear mechanical load. However, the covalent bond may destroy the integrality of sp^2^ hybridization in the microstructure of graphene so that the electrical and thermal conductivities may decrease. Given that via noncovalent modification the interactions between graphene and the modifier substances are weak which is unbenefited for load transfer, covalent modification is obviously a better choice.

In a typical oxidation-reduction method, GO is obtained from graphite via oxidation by a strong oxidant like concentrated sulfuric acid and grafted with hydrophilic functional groups, such as –OH, epoxide, –COOH. Then GO is reduced to graphene by hydrazine hydrate, hydroquinone, NaBH_4_ or other reductants with organic or inorganic functional groups [26,27]. The functional groups remain on reduced graphene render larger interlayer space and strong interfacial interaction with the polymer matrix is demanded for mechanical stress transfer [28]. Jang et al. systematically explored the implication of long-chain alkylamine functionalization of GO on electric conductivity and dispersion of GO in organic solvents. Through Hummer’s method and addition of alkylamine and hydrazine hydrate, octylamine functionalized GO exhibited optimal electric conductivity up to 180 S/m [29]. Yoo et al. obtained highly soluble functionalized GO and reduced graphene oxide (rGO) both in inorganic and organic solvents by polyetheramine (PEA) due to the amphipathicity of PEA [30]. Goods et al. synthesized covalently modified graphene with high compressive strength from GO and triethyl phosphite in the presence of LiBr. The graphene and lithium phosphate oligomers were connected through covalent phosphonate linkages and the mechanical properties could be controlled through altering the variety of metal halide [31]. Kumar et al. compounded graphene/polyurethane nanocomposites with better thermal stability than pure matrix. The key point was that p-aminobenzoic acid functionalized GO acted as the pseudo-crosslinker for composites [32]. Tang et al. applied polyetheramine to modify and reduce GO at the same time. Except for the solubility mentioned above, polyetheramine could enhance conductivity and mechanical performance of rGO/epoxy composites as well [33]. Wang et al. firstly reduced and modified GO by tea polyphenol and the material had good dispersion in both water and some organic solvents. Since tea polyphenol was environmentally friendly and had biocompatibility, the material would have promising applications in biological materials field and broaden researchers’ horizon in increasing the options of a modifier [34]. Shen et al. utilized a two-step diimide-activated amidation by 1-hydroxy-2,5-pyrrolidinedione to modify GO and the procedure was benefited for mass production. The material was amphipathic and performed long-term stability which was facilitative to yield even composites [35]. Some researchers have also studied graphene-based nanocomposites with covalent bonds between matrix and fillers which performed better mechanical and thermal properties versus the neat matrix polymer [36,37,38]. However, most methods were in two or more steps [39,40,41] with virulent modifier or reductant like hydrazine hydrate, and the processes were sophisticated. Meanwhile, in order to make the polymer matrix and graphene filler blended sufficiently, larger interlayer spaces of reduced graphene are essential and desirable. The reduction of graphene oxide with hydrazine, which is one of the most common reductants for GO, was investigated by using small organic molecules as models, and thermal treatment was beneficial for decarboxylation and dihydroxylation [42]. So the reductants consisting of hydrazine group and other functional groups were required. The authors [43] demonstrated simultaneous functionalization and reduction of GO by 4-hydrazinobenzenesulfonic acid (HBS) and the surface of rGO was grafted with an HBS molecular chain consisting of hydrophilic sulfonic acid group, which made the water solubility of graphene much more improved from 0.58 mg/mL to 13.49 mg/mL. The interlayer space of HBS-rGO was almost twice that of GO, and the aggregations was attenuated.

In this article, a one-step reduction and surface modification procedure of GO by a new reductant 3-hydroxy-2-naphthoic hydrazide (HNH) and synthesis of graphene modified polypropylene nanocomposites were prepared. The process was easy to operate and the interlayer space of the obtained HNH reduced graphene (HNH-rGO) was increased to 1.921 nm compared with 0.751 nm of GO. It was demonstrated that, as the amount of graphene filler increased, the rheological behavior of composites was changed. Moreover, surface modified graphene demonstrated a stronger viscosity improving effect than the untreated graphene.

## 2. Experimental

### 2.1. Materials

Graphene used in this study was supplied by Nanjing XF NANO Materials Tech Co., Ltd., Nanjing, China. Graphene oxide and polypropylene (PP) were purchased from Aladdin Company, Shanghai, China. 3-Hydroxy-2-naphthoic hydrazide (HNH) and all the other chemical materials were obtained from Sigma-Adrich (Steinheim, Germany). All chemicals were used without further treatment.

### 2.2. Synthesis of HNH Reduced Graphene from GO

**Figure 1:**
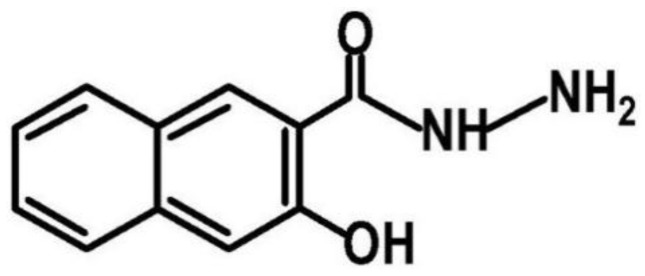
Chemical structure of 3-hydroxy-2-naphthoic acid hydrazide (HNH).

**Figure 2:**
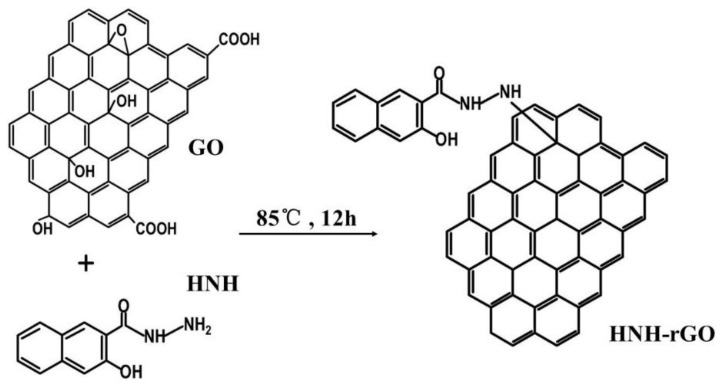
Synthesis reaction of HNH modified reduced graphene oxide (HNH-rGO) from graphene oxide (GO).

HNH was used to reduce and modify GO powder in one step and its chemical structure is showed in Figure 1. Via reduction and covalent modification reaction between the hydrazine group of HNH and epoxy group on the surface of GO, as showed in Figure 2, the surface of obtained HNH-rGO was grafted with HNH with hydroxyl groups. A typical process was described as follows. Firstly, 0.36 g GO powder and 0.46 g HNH were added into 200 mL ethanol solution with 50% ethanol and 50% distilled water. Then the mixture was ultrasonic dispersed for 20 min (45 kHz, 100 W) and stirred for 10 min. After heated at 85 °C for 5 h, the mixture was filtered with 0.22 μm filter paper and remained the filtrate. The black HNH-rGO product was obtained after purified with 50% ethanol solution to absolutely remove the remaining impurities and dried at 80 °C overnight in a vacuum.

### 2.3. Synthesis of HNH-rGO/PP and G/PP Composites

In a typical synthesis procedure of HNH-rGO/PP and G/PP composites, 30 g PP and different amount of HNH-rGO or graphene were mechanically mixed in a beaker. After mixed homogeneously, the mixture was put into a torque rheometer and stirred at 180 °C for 5 min to obtain the composites.

### 2.4. Characterization

X-ray diffraction (XRD) patterns were recorded from 3°–60° (2θ) using X-ray diffractometer (Rigaku Corporation SmartLab, Tokyo, Japan) with a Cu Kα radiation source (k = 1.5406 Å). Scanning electron microscope (SEM) observation was performed on Zeiss Merlin Compac (Oberkochen, Germany). Raman spectra were obtained on LabRAM HR800 spectrometer (Paris, France) using a 488 nm laser. X-ray photoelectron spectroscopic (XPS) characterizations were operated on Thermo Fisher ESCALAB 250Xi (Maple Plain, MN, USA). Fourier transform infrared spectrum (FTIR) measurements were carried out with the KBr pellet method on Perkin-Elmer spectrometer (Norwalk, CT, USA).

## 3. Results and Discussions

### 3.1. Characterization of HNH-rGO

**Figure 3:**
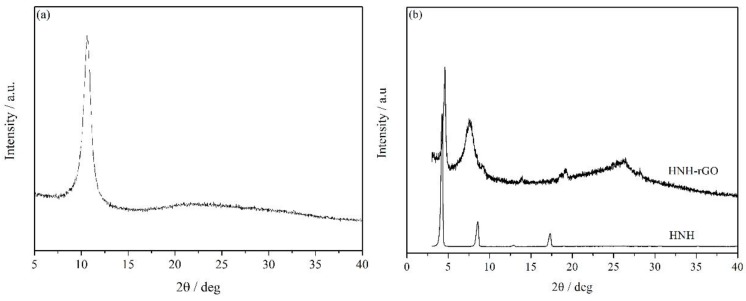
The X-ray diffraction (XRD) patterns of GO (**a**) and HNH-rGO (**b**).

The XRD pattern of GO, HNH and HNH-rGO samples were presented in Figure 3. As shown in Figure 3a, the interlayer space of GO was about 0.751 nm when calculated by the Bragg diffraction equation. As for HNH-rGO samples displayed in Figure 3b, the wide peak of graphene that appeared at about 26° represented the (002) and the peak of GO at about 11° disappeared, which indicated that GO was entirely reduced to graphene by HNH. Additionally, the interlayer space of HNH-rGO was extraordinarily enlarged to about 1.921 nm, which was almost triple the interlayer space of GO. This result was mainly attributed to the surface of HNH-rGO successfully grafted with HNH. Via covalent modification reaction, the molecular chain of HNH was grafted on the surface of graphene and inserted into graphene layers, resulting in the increase of layer interspace and decrease of Van der Waals’ force. Compared with the authors’ previous research about HBS-rGO [43], the interlayer space of rGO was enlarged by 30% from 1.478 nm to 1.921 nm mainly owing to the extended length of molecular chain of the modifier.

**Figure 4:**
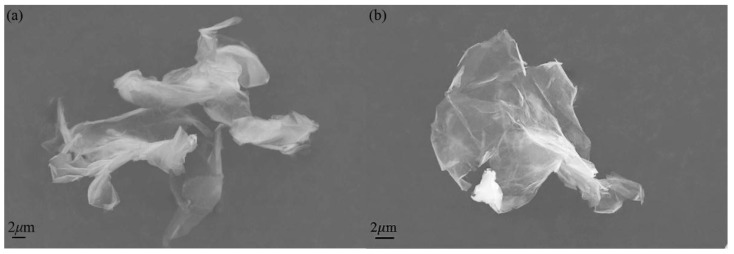
The scanning electron microscope (SEM) images of GO (**a**) and HNH-rGO (**b**).

Figure 4 displayed the morphology of GO and HNH-rGO samples. It was clear that the two samples both took on layered structure. As shown in Figure 4, GO was more inclined to get aggregations compared with HNH-rGO due to smaller interlayer space resulting in stronger Van der Waals’ force, which accorded with the XRD results.

**Figure 5:**
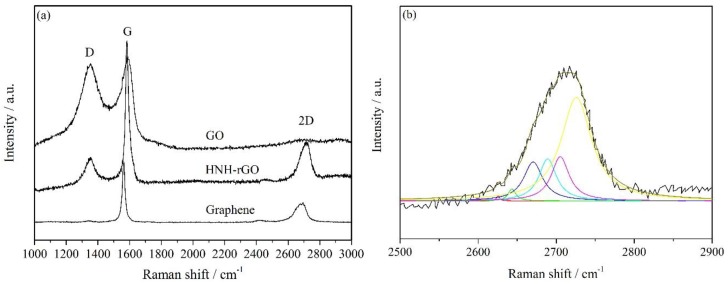
The Raman spectroscopy of G, GO and HNH-rGO (**a**) and the 2D peak of HNH-rGO (**b**).

Figure 5 showed the Raman spectroscopy of graphene, GO and HNH-rGO. As for graphene, the typical Raman scattering features of graphene are G peak, 2D peak, and D peak. The G peak (1580 cm^−1^) is due to the doubly degenerate zone center *E*_2*g*_ mode and originated from the vibration in plane of sp^2^ hybrid carbon atoms. The 2D (2700 cm^−1^) peak is the second order of zone-boundary phonons. The defects in graphene will be reflected on the D peak (1350 cm^−1^). As presented in Figure 5, the G band of HNH-rGO (1585 cm^−1^) compared with GO shifted closer to graphene (1563 cm^−1^), which indicated a successful functionalization of reduced GO and a recombination of sp^2^-hybridized graphitic network at mean time. Besides, the intensity ratio of D/G peaks that generally imply the defect concentration of graphene and the defect concentration of graphene can be calculated by: $n_{D\;}\left(cm^{-2}\right)=\left(7.3\pm 2.2\right)\times 10^{9}E_{L}^{4}\left(\frac{I_{D}}{I_{G}}\right)$ [44]. The results showed that the *I_D_/I_G_* ratio of HNH-rGO was 0.46 smaller than that of GO (0.93), so the surface defects of HNH-rGO was reduced by half and the sp^2^-hybridized degree was improved. However, the *I_D_/I_G_* ratio of HNH-rGO was a little bigger than that of graphene (0.09) and the authors’ previous research (0.29) [43], mainly due to the defects resulting from HNH groups grafted on rGO and some remaining unstable structure defects. Moreover, the intensity of G peak, the intensity ratio of G/2D peaks and the peak curve of 2D peak are usually the criterions to confirm the layers of graphene. As the layers increase, the intensity of G peak increases and the 2D peak evolves into 4 and 6 lorenz curves corresponding to bilayers and three layers, respectively [45]. In the research, the HNH-rGO was three-layer, as shown in Figure 5b, and the intensity of G peak increased compared with graphene. All the results gave adequate reasons for a sufficient reduction of GO by HNH and modification of reduced GO.

**Figure 6:**
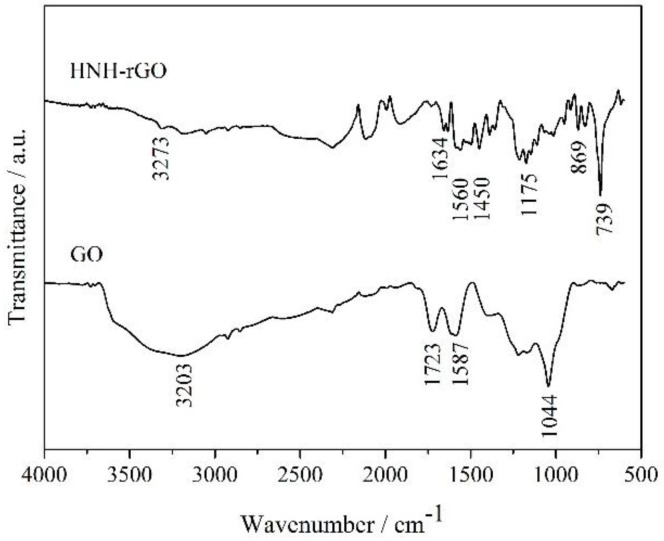
The Fourier transform infrared (FTIR) spectra of GO and HNH-rGO.

Fourier transform infrared spectrum (FTIR) is an effective method to detect organo-functional group and can give proof to the successful modificaton. As displayed in Figure 6, the characteristic transmittance bands of GO were identified, related to carbonyl stretching vibration at 1723 cm^−1^, C–O–C stretching vibration at 1044 cm^−1^, stretching vibration of aromatic C=C at 1587 cm^−1^, and the stretching vibration of hydroxyl groups was observed as a broad strong band centered at 3364 cm^−1^. After being reduced and modified by HNH, the intensities of hydroxyl groups and C–O–C stretching vibration were dramaticlly decreased as can be seen in Figure 6. This confirmed the successful reduction of GO. Furthermore, the appearance of stretching vibration of N–H at 3273 cm^−1^, carbonyl of acylamino at 1634 cm^−1^, C–N at 1175 cm^−1^ and aromatic ring at 1450 cm^−1^ gave adequate evidences to the successful modification that the molecular chain of HNH was grafted onto the surface of rGO. Besides, the peaks at 1560 cm^−1^ and 739 cm^−1^ corresponded to stretching vibration of aromatic C=C in graphene and the ortho-disubstituted aromatic ring in HNH, respectively.

**Figure 7:**
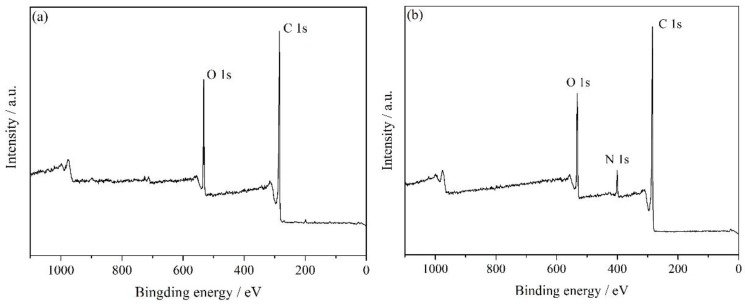

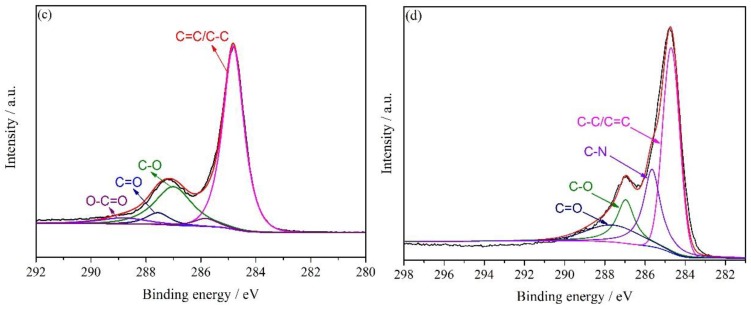
The X-ray photoelectron spectroscopic (XPS) spectra of GO and HNH-rGO: all elements survey scan of GO (**a**); HNH-rGO (**b**); C 1s survey scan of GO (**c**) and HNH-rGO (**d**).

Figure 7 showed XPS spectra of GO and HNH-rGO. In Figure 7a, GO mainly consisted of C and O elements, and the atom percentage contents were 82.81% and 17.19%, respectively. As for HNH-rGO in Figure 7b, the main elements were C, O, N, which was consistent with the materials, and the content of C and O down shifted to 77.52% and 15.52%, respectively. Given that hydrazine group was the primary pattern of N element, the content of O element in HNH-rGO except for that in HNH was calculated as 7.57% which was similar to the result of HBS [43] of 7.23%. The decrease of O content from 17.19% to 7.57% provided valid evidence for the complete reduction of GO. Figure 7c,d presented the C 1s survey scan of GO and HNH-rGO. The O–C=O totally disappeared and the intensity of C–N was obvious due to the successful reduction and modification. The C–O and C=O in HNH-rGO were mainly related to hydroxyl on naphthalene and acylamino in HNH.

### 3.2. Rheological Behavior of HNH-rGO/Polypropylene Composites

**Figure 8:**
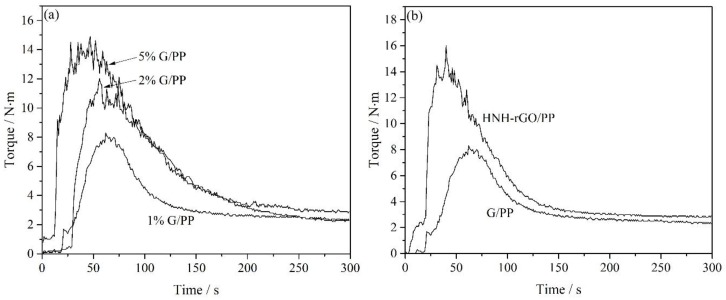
Rheology behavior of 1%, 2%, 5% graphene/PP composites (**a**); 1% graphene/PP composites and HNH-rGO/PP composites (**b**).

Figure 8 exhibited the rheology property of the composites tested on torque rheometer. The rheological behavior of graphene/PP composites with different loading of graphene was demonstrated in Figure 8a. The curves showed three stages. At first, the torque rapidly rose up to the maximum value, then rapidly reduced to a certain value and, finally, remained stable. The three stages were consistent with the glassy, elastomeric and viscous flow states of polymers from solid to molten state, respectively. With increased addition of graphene, the time before the torque of the composites reached the maximum gradually reduced and the maximum torque got higher. Graphene has incredible heat-conducting properties, so the higher amount of graphene much improved the thermal conductivity of composites. In addition, because graphene acted as a fortifier in the matrix, as the amount of graphene increased, intermolecular force became stronger and viscosity and torque was increased. Compared with graphene/PP composites, HNH-rGO/PP composites demonstrated higher torque. After HNH was grafted on the surface of reduced graphene, the interlayer space was increased so that HNH-rGO could be dispersed homogeneously and form physical crosslinking with PP more easily to make intermolecular force and viscosity further enhanced.

## 4. Conclusions

In consequence, through a one-step reduction and covalent modification method by a new reductant HNH, GO was successfully reduced and modified into HNH-rGO grafted with the molecular chain of HNH. The interlayer space of HNH-rGO increased from 0.751 nm to 1.921 nm so that the Van der Waals’ force was decreased and the agglomeration was ameliorated. The XRD result and decrease of O content from 17.19% to 7.57% provided valid evidences for the successful reduction of GO. The appearances of stretching vibration of N–H, carbonyl of acylamino, C–N and aromatic ring provided adequate reason for the successful modification. In contrast with HBS, HNH exhibited a better effect on enhancing interlayer space. HNH-rGO/PP composites performed with better rheology property than graphene/PP composites. This study revealed that HNH-rGO was a potential filler to obtain homogenous polymer nanocomposites coatings with preferable rheology behavior.
